# How best to use and evaluate Patient Information Leaflets given during a consultation: a systematic review of literature reviews

**DOI:** 10.1111/hex.12487

**Published:** 2016-09-26

**Authors:** Mélanie Sustersic, Aurélie Gauchet, Alison Foote, Jean‐Luc Bosson

**Affiliations:** ^1^ TIMC‐IMAG University of Grenoble Alpes Grenoble France; ^2^ Groupe Hospitalier Mutualiste de Grenoble (GHM) Grenoble France; ^3^ LIP/LPC2S, EA 4145 University of Grenoble Alpes Grenoble France; ^4^ Inserm CIC 1406 Grenoble Alpes University Hospital Grenoble France

**Keywords:** adherence, compliance, consultation, Patient Information Leaflet, patient's behaviour, patient's knowledge, patient's satisfaction

## Abstract

**Background:**

In the past, several authors have attempted to review randomized clinical trials (RCT) evaluating the impact of Patient Information Leaflets (PILs) used during a consultation and draw some general conclusions. However, this proved difficult because the clinical situations, size and quality of RCTs were too heterogeneous to pool relevant data.

**Objective:**

To overcome this 30‐year stalemate, we performed a review of reviews and propose general recommendations and suggestions for improving the quality of PILs, how to use them and methods for evaluating them.

**Methodology:**

We searched five databases for reviews, systematic reviews and meta‐analyses describing PILs. We drew general and condition‐linked conclusions concerning the impact of PILs. Checklists summarize criteria for quality PILs, and ways of using and evaluating them.

**Results:**

Of 986 articles found, 24 reviews were pertinent; the five oldest considered the impact of PILs irrespective of the condition the patient consulted for; the 19 more recent ones mostly addressed precise clinical situations.

**Discussion:**

Whatever the clinical situation, PILs improve patients' knowledge and satisfaction. For acute conditions, in the short‐term PILs also improve adherence to treatment. For chronic diseases, invasive procedures or screening situations, their impact on adherence varies depending on the context, how the PILs are given and the invasiveness of the intervention.

**Conclusion:**

PILs are considered to be very useful, especially for acute conditions where the patient is the first to suffer from lack of information. We propose checklists for writing, designing, using and evaluating PILs in RCTs to enable comparisons between different studies.

## 1 Introduction

Since the 1970s, various authors have investigated the use of Patient Information Leaflets (PILs)^1^, ^2^, ^3^, ^4^ and have suggested that they are helpful for patients, particularly as they improve recall of what was said during the consultation.^2^, ^5^, ^6^ Although more and more information is available through the Internet, patients continue to ask for more written information.^7^, ^8^ However, the availability of PILs does not necessarily guarantee access to quality information tailored to the needs of each patient.^9^, ^10^, ^11^, ^12^

In the 1990s, Dixon and Park underlined the importance of developing recommendations to improve the quality of PILs.^13^ Although health‐care institutions and the research community have developed guidelines to help create PILs,^6^, ^14^, ^15^, ^16^ the use of these is rarely reported by the medical and research community. Other authors have looked at how PILs are used in everyday practice.^4^, ^16^, ^17^ There is general agreement that PILs should be handed out by the physician at an opportune moment during the consultation,^6^, ^18^ should target patient expectations^10^, ^16^, ^18^, ^19^, ^20^, ^21^ and that the form should take into account the patient's preferences.^18^, ^22^ The PILs should back up what the physician says^6^, ^16^, ^17^, ^18^ but should in no way be a substitute for oral information, preferred by the majority of patients.^4^, ^16^, ^17^, ^18^ However, research protocols generally do not take these considerations into account: the PILs is sometimes distributed by the nurse,^23^, ^24^ sometimes by a pharmacist,^25^, ^26^ by a clinical research assistant or by another person. Sometimes it is sent by email^27^ or by post^28^ in spite of the fact that informing the patient is now considered by medical institutions as the physician's responsibility.^14^, ^29^ In view of problems such as these, the most recent review of “generalist” literature on PILs (i.e. not specific to a given clinical situation) conducted in 1998^16^ stressed the need for further research in general, and in particular using randomized clinical trials (RCTs).

Since then, many groups have used RCTs to assess the impact of PILs in specific clinical situations, such as in chronic illness,^22^ contraception^30^ screening,^31^ chest pain in the emergency room,^23^ preparing for surgical interventions^9^ or in consultations with a primary care physician.^1^, ^2^, ^32^ However, problems of heterogeneous research protocols remain, both in the choice of primary outcome and in the main measurement technique, resulting in conclusions that are sometimes contradictory.^32^ Studies concerning a given condition have been reviewed within the appropriate field.

We have attempted to summarize the diverse reviews, both general and specific to given conditions (literature reviews, systematic reviews and meta‐analyses), made to date. We clarify the impact of PILs by evaluating their effect on main outcomes, and specify their prescription according to condition and terms of use. In addition, we propose a checklist for writing, designing and using PILs with recommendations for the standardization of research protocols that assess PILs.

## 2 Methods

### 2.1 Literature search and study selection

We systematically searched PubMed, Embase, Cochrane Library, Web of Science and PsychInfo for original articles using the following Mesh terms: “handout”, “leaflet”, “booklet”, “pamphlet”, “flyer”, “folder”, “brochure”, “written patient information”, all synonyms AND “patient”. Filters used were “meta‐analyses”, “literature reviews”, “systematic reviews” without temporal or language restrictions. By chance, all the reviews found were in English. After merging the results from the different search engines, duplicate publications were removed. The relevant free access articles and those available through our university or national research organisations were recovered. Otherwise, if the title or abstract were relevant, the authors were contacted by email and if there was no response, their articles were ordered from the publisher. Review articles cited in extracted articles were also used. For our purposes, the PILs should contain information on the disease for which the patients consulted. Hence, we excluded reviews of leaflets aimed at multifaceted studies in which no leaflet‐specific effect could be extracted, reviews concerning decision aids, or on patient consent documents and reviews of patient empowerment tools.

To extract all relevant articles, two primary care physicians (MT and JT) separately assessed all articles found by the search engines using the titles and abstracts. Only articles selected by both were retained, and when they disagreed, the abstracts were reassessed by one and checked by the other. Selected abstracts were discussed with an expert (MS) in PILs and disagreements resolved by consensus. We checked the reference lists of the selected reviews for additional relevant publications.

### 2.2 Data extraction

Data were extracted from the full texts of the selected reviews by the two primary care doctors working independently. A standardized form was used to record the relevant characteristics of the included reviews: methodology, condition studied, population, intervention, outcome measures, study quality, the number of articles included in the review, the total number of patients (if available) and main conclusions. We (MS and AG) checked the concordance rate between the doctors and resolved any disagreement by consensus (MT, JT, MS and AG).

**Figure 1:**
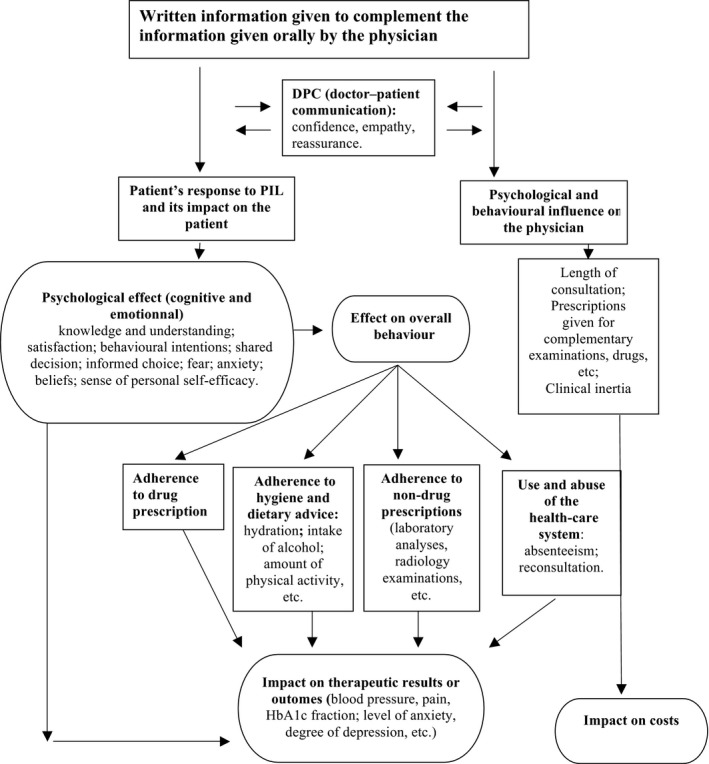
Theoretical model analysing the impact of Patient Information Leaflets on patients and their physician using a multidisciplinary phenomenological approach

To anticipate and resolve disagreements regarding the terminology for the different outcomes (for example, for some authors “adherence” relates only to drug adherence,^33^ for others, it includes respect of the drug regime, lifestyle changes and changes in diet^34^), we built a framework using a multidisciplinary phenomenological patient‐centred approach by grouping outcomes according to the type of impact (Fig. 1). Our theoretical model includes three types of impact on the patient: on the psyche of the patient (cognitive and emotional), on behaviour (e.g. adherence) and on therapeutic results; and one type of impact on doctor behaviour (prescribing). The impact of doctor–patient communication holds a special place because it involves both the physician and the patient.

Our model was based on the work of Garner et al.,^35^ Downie et al.,^36^ McDonald et al.^34^ and all the literature on the impact of PILs. For Garner, the effectiveness of an information leaflet is evaluated by its emotional, cognitive and behavioural impact in terms of the doctor's initial intentions. Downie et al. describe the impact of information successively on patient's knowledge, their intention to make changes and their behaviour. Finally, McDonald et al. define adherence as the patient's behaviour in dealing with drug and/or non‐drug prescriptions, in following lifestyle advice and in attending consultations. We added the category “therapeutic outcomes” in line with numerous articles and to reflect behavioural consequences.^6^, ^16^, ^17^

### 2.3 Methodological quality assessment

We evaluated the methodological quality of included reviews using the criteria of the Cochrane Handbook for Interventional Systematic Reviews and the Preferred Reporting Items for Systematic Reviews and Meta‐Analyses (PRISMA). Each review article was rated as “low quality”, “good quality” or “very good”.

## 3 Results

### 3.1 Article selection

**Figure 2:**
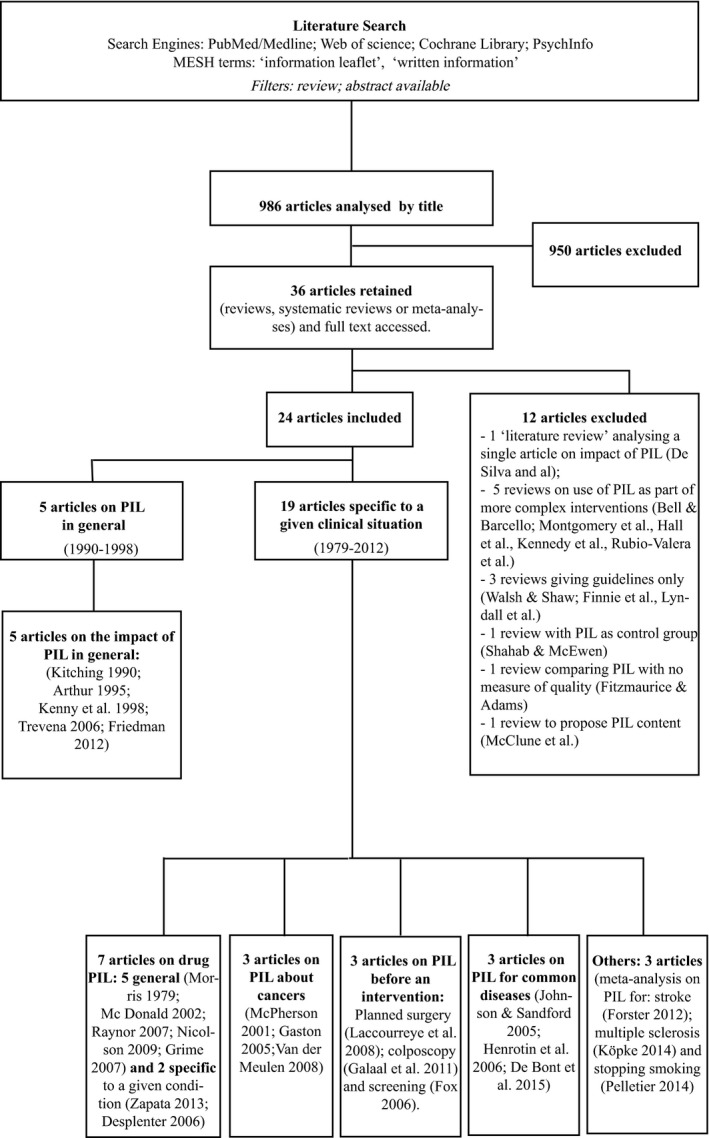
Literature search flow chart

Our search identified 986 unique records of which 950 did not meet our inclusion criteria following the screening of titles and abstracts. Of the remaining 36 articles, after evaluation of the full text, only 24 met the inclusion/exclusion criteria (Fig. 2). The rate of concordance for data extraction between the two doctors was 95%.

### 3.2 Review characteristics

Between 1990 and 2012, five literature reviews evaluated the “general” impact of PILs on patients, whatever the medical conditions: the oldest three focused exclusively on PILs^5^, ^6^, ^16^ while the two more recent looked at the impact of any form of information on patients.^9^, ^20^ Since 2012 (up to August 2015), all the reviews we found were specific to a given situation or condition. Seven reviews looked at information on drugs (including one on contraceptives and one on drugs for psychiatric disorders), three reviews concerned PILs for cancer patients, three were on PILs intended to be given before a screening examination or surgery, three were about common acute conditions and three on chronic diseases. The main characteristics of all these reviews (outcome measures, etc.) are summarized in Table 1. According to the PRISMA checklist for study quality, 10 were of very good quality, nine were good and five were of poor methodological quality.

**Table 1:** Summary of reviews selected for analysis

Author(s), year	Type	No. of studies	No. of patients	Population/condition	Interventions	Methodologic quality^a^	Impact of PILs
Morris and Halperin, 1979^21^	L	/	/	Various conditions	Drug PILs vs nothing or PILs as part of more complex interventions.	Poor	Positive effect on knowledge. Indeterminate effect on side‐effects. No effect on adherence.
Kitching, 1990^6^	L	30	/	All kinds of patients	/	Poor	Effect on knowledge Indeterminate effect on adherence and therapeutic outcomes
Arthur, 1995^5^	L	/	/	Literature review	PILS vs leaflet oral information or oral information alone	Poor	Increases knowledge and satisfaction but not adherence.
Kenny et al., 1998^16^	L	/	/	All kinds of patients	PILs	Poor	Reduction in anxiety, pain, depression and the number of re‐consultations. Improvement in adherence, knowledge, satisfaction. PILs can be an alternative to the prescription of drugs.
McPherson et al., 2001^10^	S	10 RCT	/	Cancer	PILs, audiotapes, audiovisual aids or interactive media	Good	Positive effect on knowledge, symptom management, satisfaction, health‐care utilization and affective states, although effect on psychological scores
McDonald et al., 2002^34^	S	33 RCT	/	Acute and chronic disease	PILs, PILs as part of more complex interventions	Good	Indeterminate effect on adherence (urine test, telephone interview, pill count, patient self‐report) and clinical treatment outcomes (throat culture, breath test, blood pressure, adverse effects)
Johnson and Sandford, 2005^17^	S	2 RCT	320	Acute thermal injury, otitis media	PILs + verbal information vs verbal information only	Good	Positive effect on knowledge and satisfaction
Gaston and Mitchell, 2005^7^	S	12 RCT, 3 RT, 32 studies	/	Advanced cancer	Interventions to improve information giving or to improve participation in treatment decisions (PILs, many other kinds of intervention)	Good	Positive effect on anxiety, satisfaction, knowledge and understanding. Non‐effect on psychological outcomes
Henrotin et al. 2006^39^	S	11 RCT	8558	Low back pain	PIls, video programme, multimedia campaign, Internet‐based information	Good	Positive effect on knowledge. No effect on absenteeism. Indeterminate effect on pain, degree of disability or health‐care use
Fox, 2006^31^	L	9 RCT	/	Various screening programmes	PILs vs nothing, PILs as control compared with other intervention, PILS is a part of more complex interventions	Very good	Positive effect on knowledge. No effect on informed choice
Trevena et al., 2006^19^	S	10 S + 30 RCT	/	Case scenario: PSA screening for prostate cancer	Decision aids/PILs/Videos/Websites/Tailored computer programs/Verbal advice/Structured counselling compared to no tool or other tools	Good	Positive effect on understanding and knowledge in health care
Desplenter et al., 2007^7^	S	17 RCT	/	Mental illness	PILs alone, PILs as control, PILs as part of more complex interventions	Good	Positive effect on adherence, knowledge. No effect on satisfaction, frequency of side‐effects, relapse, readmission rates, symptoms or quality of life
Raynor et al. 2007^4^	S	70 studies + 43 RCT	/	All kinds of patients	Drug PILs vs nothing, verbal information or comparison of various PILs	Very good	Indeterminate effect on knowledge. No effect on adherence to long‐term therapy or change in the reporting of side‐effects
Grime et al., 2007^18^	S	27 studies	/	Patient with drug prescriptions	Drug PILs	Good	The policy initiative and adherence studies reported that most patients were generally positive about written information received
Laccourreye et al., 2008^9^	L	/	/	Surgery	PILs delivered during a consultation before programmed surgery	Poor	Indeterminate effect on recall and anxiety
Van der Meulen et al., 2008^22^	S	8 RCT, 1 CT, 1 RT	/	Cancer patients	Audiotaped consultations with and without PILs; and other interventions	Very good	PILs help recall of information
Nicolson et al., 2009^46^	S	25 RCT	4788	Patients with drug prescriptions for chronic or acute conditions	Interventions using drug PILs	Very good	Positive effect on knowledge. Indeterminate effect on attitudinal and behavioural outcomes
Friedman et al., 2011^20^	S	23 S + M	/	Chemotherapy, type 2 diabetes, asthma, analgesia	Teaching interventions (traditional lectures, discussions, simulated games, computer technology, PILs, audiotapes, videotapes, verbal, demonstration) vs standard care (control) or vs another teaching intervention	Very good	Positive effect on knowledge, anxiety, satisfaction
Galaal et al., 2011^40^ (updated version, 1st version in 2007)	M	6 RCT	886	Colposcopic examination	PILs vs nothing, PILs as control group (compared to other interventions), PILs as part of more complex interventions	Very good	Positive effect on knowledge, patient quality of life, psychosexual dysfunction. No effect on anxiety
Forster et al., 2012^38^	S	21 RT	2289	Stroke and transient ischaemic attack	PILs vs usual care/PILs and another intervention vs the same intervention	Very good	Increases knowledge but no effect on anxiety and depression
Zapata et al., 2013^30^	S	5 studies + 3 RCT	/	Contraception	Exposure to some written material (e.g. patient package inserts, brochures)	Good	Positive effect on knowledge. No effect on women's intended actions after missing pill
Pelletier et al., 2014^54^	S	/	Smoking cessation	PILs vs other interventions	Very good	Positive effect on satisfaction. No effect on cessation (except for two studies using PILs + motivational interviewing‐based interventions)
Köpke et al., 2014^55^	S	10 RCT	1314	Multiple sclerosis	PILs, educational programmes or lectures, audiovisual aids, Web‐based learning, decision support tools, personal information	Very good	Positive effect on knowledge. No adverse events reported. No effect on decision making or quality of life
De Bont et al., 2015^32^	S	8 studies (7 RCT + 1 study)	3407	Respiratory tract infections, conjunctivitis, urinary tract infections, gastroenteritis and tonsillitis	PILs or interactive booklet vs no intervention	Very good	Positive effect in reducing antibiotic prescriptions by GPs, antibiotic use by patients and their intention to reconsult for future similar episodes of illness. Indeterminate effect on reconsultation rates

S, systematic review; L, literature review; M, meta‐analysis; RCT, randomized controlled trial.

a According to PRISMA checklist.

### 3.3 The impact of PILs on the psyche, cognitive and emotional state of the patient

*The dimensions we studied were patient's understanding, knowledge, recall of what was said, informed choices, satisfaction, emotional reaction, shared decision making and beliefs*.

Of 24 reviews, 18 described the impact on knowledge (75%), 11 the impact on satisfaction (46%), seven the impact on mood (anxiety) (29%), four the impact on decision making, two the impact on decisional conflict and one the impact on beliefs.

Across all clinical situations, PILs have a major impact on patients' knowledge.^5^, ^6^, ^16^, ^17^, ^37^, ^38^, ^39^ This impact on knowledge is improved when the PILs are concise but precise,^1^, ^30^, ^40^ sufficiently detailed (disease, dietary and lifestyle advice, expected and adverse effects of drugs, etc.),^5^, ^18^ include graphical presentations,^5^, ^20^, ^30^ are written in the active voice^14^, ^38^, ^41^ and solicit reflection on the part of the patient by posing questions.^19^, ^42^ However, the extent of this impact also depends on the clinical context and/or invasiveness of the intervention (improvement in knowledge from 18% to 57% depending on the case)^9^, ^31^ and the timing of receiving the information.^4^, ^6^, ^9^, ^42^ For example, in the case of cancer, written information in the form of new patient information booklets or packages improved patient knowledge and reduced confusion especially if it was provided to the patient before their first clinical appointment rather than at the first appointment.

Before a surgical procedure, 95% of patients want to be informed about the associated risks^9^. However, the information is misunderstood 25% of the time, and patients deem the information inadequate (15% of leaflets) or perceive the leaflet as simply a way to protect the judicial rights of the surgeon.^9^ Conversely, immediately after surgery may be an inappropriate moment to give written information because at this point patients seem to retain very little of the information provided.^9^ Few studies have evaluated the impact of PILs on knowledge beyond 15 days after reception.^9^ However, it is agreed overall that PILs improve patient satisfaction.^5^, ^10^, ^17^, ^20^

Many other psychological dimensions (sense of personal self‐efficacy, decisional conflict, doctor–patient communication, empathy, trust, listening, etc.) have also been explored over the past 20 years, but as definitions of outcomes and the tools used to assess them vary between teams and disciplines, we were unable to draw clear conclusions. For example, a common item called “decision making” is used in three different questionnaires for three different purposes (Physician Patient concordance,^43^ Control Preference Scale^44^ and Sharesd Decision‐Making Questionnaire^45^).

### 3.4 The impact of PILs on patient behaviour

*The dimensions studied were adherence (nowadays the term adherence is preferred to compliance) to treatment or lifestyle, return to work, rates of reconsultation, absenteeism and participation in screening*.

Through PILs, physicians especially aim to improve the adherence of patients to treatment but patients want to be informed even if they do not ultimately follow recommendations.^4^, ^18^, ^30^ Patients look to written information to help their decision making, starting with as to whether to follow a particular treatment or not.^4^ Sometimes an informed choice can lead a patient to not take a drug.^4^, ^21^, ^30^ The impact of PILs on behaviour depends on the clinical situation.^9^, ^31^, ^32^ For common diseases such as acute otitis media, burns,^17^ low back pain,^39^ PILs improve treatment adherence^6^, ^34^, ^37^ and/or adherence to lifestyle and dietary guidelines.^10^, ^16^, ^39^ Many women who miss taking contraceptive pills may choose not to follow the recommended actions despite clear instructions.^30^ All in all, PILs do not guarantee that behavioural changes will be made.^5^, ^21^, ^30^, ^31^ Some authors state that improving the behaviour of the patient is not the role of PILs and that the patient's choice must be respected.^4^, ^30^, ^46^

When used prior to surgery, the PILs can sometimes lead patients to refuse surgery (3.2% to 14.6% depending on the procedure).^9^ Similarly, in the context of screening, PILs can lead to acceptance or potentially to refusal to undergo an examination. For example, one study showed that PILs could improve consent to screening with a prostate‐specific antigen test but did not improve the acceptance of screening involving a digital rectal examination.^31^ Another concluded that before a colposcopic procedure they were useful for obtaining patient consent.^40^ Nevertheless, in a review of studies on screening programmes for several conditions, Fox^31^ found that PILs had no clear effect on screening uptake. Due to their potential influence in decisions, PILs must be thoughtfully provided and used with care.

For common conditions, PILs improve adherence to medication and advice.^32^ PILs also decrease the number of repeat visits to the primary care physician.^32^ For example, in the case of lower back pain,^39^ the precise instructions given by the leaflet boosted patient confidence, and improved beliefs in the effectiveness and adherence to short‐term exercises but did not improve the rate of attendance at appointments.

In the context of chronic conditions, the concept of clinical inertia has recently appeared in the literature. This refers to inappropriate behaviour documented in evidence‐based studies in given clinical situations (diabetes, hypertension, dyslipidaemia) and focuses on the determinants from both the patient's perspective and the physician's perspective.^42^ Aujoulat et al. introduced the concept of behavioural kinetics in adhesion to treatment. Here, depending on the patient, the context, the pathology and/or the physician, the patient's behaviour can change over time. Thus, a single endpoint measurement for patients with chronic diseases may fail to detect inappropriate behaviour.^42^

### 3.5 Impact on the results of therapy

A multitude of parameters (blood pressure, pain, anxiety and depression levels, quality of life, laboratory results, etc.) can inform therapeutic results. Except for anxiety, depression and pain, the criteria are usually specific to a given clinical situation, limiting the comparability of results between studies. Some examples include the following:

1. For cancers, PILs diminish levels of anxiety.^7^ It has been noted that for cancer in particular, information must be tailored to the needs of the individual patient to achieve better outcomes.^10^, ^20^
2. Prior to an invasive procedure, some authors find that PILs can increase anxiety,^9^ while others find they have no effect.^40^
3. Drug PILs do not increase the occurrence of side‐effects^37^, ^46^ and can even reduce them.^10^
4. For patients who had suffered a stroke or TIA, Forster evaluated the impact of all types of information (PILs, information booklets, videos, educational sessions or reading lists) from 21 RCTs. The information was classified into two categories: active (participation of subjects in planification of follow‐up, consolidation) or passive. The active information reduced levels of anxiety and depression, but without reducing the number of cases of depression. In contrast, passive information increased anxiety.

### 3.6 Impact on physician's behaviour

Patient Information Leaflets should be nuanced, distributed thoughtfully and personalized by the physician during the consultation. They must be hand delivered and must be considered in the same way as a medical prescription. Three of four studies presenting data on the prescription of antibiotics in primary care showed significant reductions in the number of prescriptions for groups receiving leaflets.^16^, ^32^ Among the reviews we studied, only De Bont et al.^32^ looked at the physician's prescribing behaviour, despite the fact that their behaviour has a direct impact on the patient in the quality of care (reduction in the number of redundant prescriptions) and on public health (through costs incurred). There is a lack of research into this aspect that merits greater study. For long‐term treatments, physicians should consider using other educational tools to supplement PILs.

## 4 Discussion

The principle targets of PILs are drug treatments,^4^, ^18^, ^46^ invasive procedures (such as surgery or colposcopy,^9^, ^40^ screening^31^ and cancer.^7^, ^10^, ^22^ Very few articles concern acute pathologies^17^ or general medicine.^32^

### 4.1 Towards the standardization of PILs

In RCTs evaluating PILs, the authors rarely question the quality of the document being tested. Yet PILs are heterogeneous both in terms of format and content. They can go from a single page^2^ up to 3, 4, 6, 28 or even 45 pages, although several studies^1^, ^30^, ^40^ emphasize the superiority of a short format. Although many guidelines are available, few authors cite any of those used nor justify their choices (e.g. choice of rubric, headings and subheadings; font; sources; whether they have checked the readability of the text, the level of the target patients' health literacy, etc.) Also, few studies compare the effectiveness of different styles of writing.^1^ The impact of different contents, styles and formats for the same clinical situation^40^ or close clinical situations^2^, ^19^, ^23^ is rarely studied, which makes difficult a comparison of PILs' effectiveness. The results of an evaluation of PILs also depend on the target clinical situation^9^, ^31^, the method of distribution^16^ and the design of the RCTs evaluating it. The challenges of doctor–patient communication are not the same for acute situations, chronic diseases, screenings or prescription of contraceptives. Handing out an information leaflet should not be considered without taking into account the profile of the patient and in ensuring good doctor–patient communication.^4^, ^18^, ^19^, ^20^ Furthermore, the physician should ensure that the patients actually wants PILs: some will not read them or will immediately discard them.^18^ Not all patients want written information,^18^ but those who do want sufficient detail to meet their needs. PILs should always be accompanied by oral information^6^, ^16^, ^17^, ^18^ for which they are not a substitute.^4^ The physician should customize the leaflet, highlighting the important points by hand during the consultation^39^ and treating it with the same importance as a prescription. Taking into account the above observations, we have drawn up a simple checklist for producing PILs (Table 2).

**Table 2:** Checklist for quality Patient Information Leaflets (PILs) according to the current literature

**Contents of PILs**
Based on the latest evidence‐based medicine^14^, ^15^, ^16^
Declares the objectives of the PILs (writer's intention)^35^
Explains causes, consequences, the usual course of the condition/disease^14^, ^15^
Explains the benefit/risks of a treatment, if any^4^, ^9^, ^41^
Gives advice on what to do if a dose is missed: conduct to take^30^
Advice on who, when and where to reconsult^2^, ^15^, ^22^
Advice on “what to do”: lifestyle recommendations, surveillance^4^, ^46^, ^49^
Takes into account the patient's needs according to the literature^18^, ^19^
Written so that it personally addresses the reader, targeted, culturally appropriate^35^, ^41^, ^50^
Contains easy‐to‐understand illustrations, diagrams or photographs^20^, ^30^, ^41^, ^49^
Names the person who wrote the leaflet and their position
States date of writing and/or last update^14^, ^15^, ^41^
Gives references to sources of the information with dates^14^, ^41^
Avoids advertising or pharmaceutical brand names, uses generic names^41^
**Design of PILs**
Favours patient interaction through questions^19^, ^50^
Short format^14^, ^15^, ^51^
Layout of information structured, presented in a logical order (paragraphs and titles)^14^, ^19^, ^41^
Not too compact, simple presentation, avoiding colour overload in drawings and boxes^15^
Simple vocabulary (words or group of words)^14^, ^35^, ^41^
Simple syntax (i.e. short sentences and active tense, active sentences)^14^, ^29^, ^41^, ^52^
Standard font (Arial, Times) avoiding small size (10 minimum)^14^, ^15^
Use of % to express frequencies, especially for risk perception^19^, ^52^
Contains a space to make notes^41^
**Other properties**
Readability verified using a standard test^14^, ^15^, ^35^
Critically read by at least two physicians in the discipline^14^, ^15^, ^50^, ^53^
Critically read by at least two potential users to test comprehension^14^, ^15^, ^35^
Available in electronic format to facilitate storage, update and traceability of use^15^, ^16^
Freely available online^15^, ^16^
Mechanism for regular update of the information and installation of literature monitoring^14^, ^15^
Planned evaluation of PILs in quality RCT^12^, ^14^, ^15^

### 4.2 Towards the standardization of research protocols evaluating PILs

When examining data from the literature, it is difficult to know whether the lack of impact of a patient information leaflet is related to the quality of the leaflet itself, the way it was used, the precise clinical situation in which it was tested or the quality of the research protocol. To overcome this impasse, it seems essential that researchers use consensually accepted standardized tools, including procedures for drafting PILs (such as those recommended here) (Table 2), instructions for the manner in which they are used or handed out and similar research protocols (Table 3). In the past, when PILs have been evaluated, the research protocols varied considerably, making comparisons between studies and meta‐analyses difficult to perform. In particular, the choice of primary outcome and its measurement are critical. A previously validated scoring system should be used and the method of completing it carefully considered (patient self‐assessment, interviews etc.). The timing of the main outcome measurement^9^ and whether a single or repeated measures are made will influence the results. However, many studies do not take this into account and attempt to measure the impact of a leaflet only a few days, weeks or months after giving it out. Most studies evaluate only one leaflet.^1^, ^3^, ^24^, ^47^, ^48^ Studies that evaluate several have created their own scores for each leaflet, also restricting the comparability of results from one study to another. While for some dimensions such as anxiety, depression or pain, validated scores allow their extent to be assessed, others, like knowledge, behaviour or doctor–patient communication still have no validated generic score to date. Despite exhaustive literature, it remains difficult to produce a synthesis of the data or to define a threshold of effectiveness of PILs by dimension and clinical situation. These endpoints are nevertheless essential in the evaluation of any PILs. There is a need to develop generic scoring systems independent of the pathological context so as to allow comparison between studies.

**Table 3:** Points to consider when evaluating Patient Information Leaflets (PILs)

Type of PILs	PILs written and designed according to a defined methodology and/or complying with the guidelines (see Table 2)^12^, ^14^, ^15^
Way of using PILs	Hand delivered at the same time as verbal information (or, if sent prior to a consultation, by post or email, at least read together with the physician during the consultation)^15^, ^16^, ^46^
	Tailored/customized according to the patient's profile by the physician during the consultation (e.g. by underscoring certain items)^10^, ^11^, ^12^, ^17^, ^18^
	Given at an opportune moment during the consultation^9^, ^18^
	Given only if the patient wants PILs^18^
Study design	Randomized allocation of patients (or cluster randomization) to PILs or a control group^14^, ^15^, ^29^
	Single blind because the physician has to go through the PILs with the patient
	Control group without PILs (oral information alone)
Outcomes	Primary outcome using one previously validated score or measure
*Acute conditions*	
Impact on patient	Outcomes using one or more of the main outcome measures commonly used in RCT
Psychic and cognitive impact	Test of comprehension/knowledge of condition
	Satisfaction
Behavioural impact	Behaviour/adherence to treatment and to advice according to the objectives of the PILs (writer's intention)
	Reconsultation rates
Therapeutic outcomes	Pain
	Depression
	Anxiety
Impact on physician	Number of drugs prescribed
	Number of examinations or laboratory tests prescribed
Impact on both patient and physician	Doctor Patient Communication effectiveness
*Chronic conditions*	Quality of life
	Relapse
	Clinical criteria (e.g. blood pressure)
	Laboratory criteria (e.g. blood glucose)
Appropriate timeline of measurement(s)	For an acute pathology/screening/surgery: 0 and 7–10 days after consultation
	For a chronic disease or long‐term prescription (except cancer); D0; D7–10; M1; M3; M6; (±M12 or M24)
Questionnaires	Use validated questionnaires
	By phone or patient self‐assessment if possible, with well‐posed questions aimed at honest replies
Investigator	Assessment of outcome by blinded doctor or CRA

## 5 Conclusion

When well written and used at the appropriate time, PILs can improve patients' knowledge and/or patients' satisfaction whatever the clinical situation and induce better adherence to treatment, to diet and to lifestyle advice, especially in the short term. The main context for the use of PILs are acute common conditions, where the patient is the first to suffer from lack of information or poor recollection of what the physician said. For chronic diseases, invasive procedures or screening situations, the impact on behaviour depends largely on the type of clinical situation and the invasiveness of the intervention, and a little less on the quality of the PILs, the manner and the moment in which it is given.

This review of reviews provides a picture of the impact of PILs on patients irrespective of the condition, while also considering specific situations. We propose a summary of recommendations for (i) writing quality PILs, (ii) their appropriate use in routine practice taking into account the clinical context and (iii) their evaluation in clinical research. It remains for research teams to work on the elaboration of generic scores independent of the clinical situations so as to allow better comparison between PILs and to set clear guidelines for their usage.

## Funding

This work had no specific funding.

## Conflict of Interest

None of the authors has a conflict of interest to declare.
